# Performance and Long-Term Stability of Pd/PSS and Pd/Al_2_O_3_ Membranes for Hydrogen Separation

**DOI:** 10.3390/membranes4010143

**Published:** 2014-03-06

**Authors:** Simona Liguori, Adolfo Iulianelli, Francesco Dalena, Pietro Pinacci, Francesca Drago, Maria Broglia, Yan Huang, Angelo Basile

**Affiliations:** 1 Institute on Membrane Technology of Italian National Research Council (ITM-CNR), c/o University of Calabria, Cubo 17/C, Rende (CS) 87036, Italy; E-Mails: s.liguori@itm.cnr.it (S.L.); a.iulianelli@itm.cnr.it (A.I.); 2 Chemistry and Chemical Technologies Department, University of Calabria, Cubo 15/D, Via P. Bucci, Rende (CS) 87036, Italy; E-Mail: dalena.ch@gmail.com; 3 RSE S.p.A., Via Rubattino 54, Milano (MI) 20134, Italy; E-Mails: pietro.pinacci@rse-web.it (P.P.); francesca.drago@rse-web.it (F.D.); maria.broglia@rse-web.it (M.B.); 4 State Key Laboratory of Materials-Oriented Chemical Engineering, College of Chemistry & Chemical Engineering, Nanjing University of Technology, Xin-Mo-Fan Road 5, Nanjing 210009, China; E-Mail: huangy@njut.edu.cn

**Keywords:** palladium composite membrane, porous stainless steel, ideal selectivity, hydrogen separation

## Abstract

The present work is focused on the investigation of the performance and long-term stability of two composite palladium membranes under different operating conditions. One membrane (Pd/porous stainless steel (PSS)) is characterized by a ~10 µm-thick palladium layer on a porous stainless steel substrate, which is pretreated by means of surface modification and oxidation; the other membrane (Pd/Al_2_O_3_) is constituted by a ~7 µm-thick palladium layer on an asymmetric microporous Al_2_O_3_ substrate. The operating temperature and pressure ranges, used for studying the performance of these two kinds of membranes, are 350–450 °C and 200–800 kPa, respectively. The H_2_ permeances and the H_2_/N_2_ selectivities of both membranes were investigated and compared with literature data. At 400 °C and 200 kPa as pressure difference, Pd/PSS and Pd/Al_2_O_3_ membranes exhibited an H_2_/N_2_ ideal selectivity equal to 11700 and 6200, respectively, showing stability for 600 h. Thereafter, H_2_/N_2_ selectivity of both membranes progressively decreased and after around 2000 h, dropped dramatically to 55 and 310 for the Pd/PSS and Pd/Al_2_O_3_ membranes, respectively. As evidenced by Scanning Electron Microscope (SEM) analyses, the pinholes appear on the whole surface of the Pd/PSS membrane and this is probably due to release of sulphur from the graphite seal rings.

## 1. Introduction

In recent years, the rise in the request for hydrogen in the petrochemical industries as well as for supplying fuel cells has led to ever increasing attention on the development of new and alternative methods for hydrogen separation/purification [1].

In this context, dense palladium membranes have received growing consideration owing to their complete selectivity towards hydrogen permeation with respect to all other gases. Indeed, an extensive literature has been reported on thick Pd unsupported membranes, which have been utilized and applied for producing high-grade hydrogen, although characterized by low permeability [1,2,3]. Recently, in order to enhance the hydrogen permeability, research has been focused on palladium thickness reduction, making possible simultaneously high mechanical resistance. Currently, the studies of scientists in this area are mainly devoted to the development of Pd-based composite membranes characterized by a thin metallic layer deposited onto porous substrates, showing such benefits as high hydrogen permeability, selectivity towards hydrogen permeation, and mechanical resistance [4,5,6,7]. Typical substrate materials for composite membranes are stainless steel and ceramics [8,9,10,11,12]. Stainless steel sintered porous supports are particularly suitable owing to good weldability and mechanical strength. They seem to be more convenient with respect to ceramic substrates for integration with Pd-based layers because of their thermal expansion coefficient being similar to Pd metal [13,14]. However, a porous stainless steel (PSS) support shows some drawbacks such as a high rough surface and a large and wide pore size, which cause some problems during membrane manufacture. Moreover, the long-term stability of these aforementioned composite membranes is affected by intermetallic diffusion, which occurs when the metallic layer is directly in contact with the porous support [15]. In order to avoid this phenomenon, different approaches, based on the use of an intermediate layer between metal and support, have been studied by various authors [16,17,18,19]. For instance, Shu *et al*. [16] have used an ultrathin (0.1 μm) intermediate layer of titanium nitride as diffusion barrier between a Pd/Ag alloy film and a PSS substrate. Nam and Lee [17] proposed silica layer utilization also for improving the structural stability of the membrane. Ma *et al*. [18] created an intermediate layer by *in situ* oxidation of the porous support. Recently, Wei *et al*. [19] reported an effective method for modifying the PSS substrate by using a pencil coating, forming a graphite layer as a diffusion barrier.

On the contrary, ceramic supports show some benefits, being chemically inert and stable over a wide temperature range [20,21]. Moreover, their structure presents essential properties for manufacturing defect-free membranes with thin metallic layers such as small pore sizes with uniform distribution. Nevertheless, in order to realize ceramic supports showing these characteristics, numerous coating and sintering steps are necessary with a consequent cost increase [22]. Therefore, low-cost supports characterized by some defects are usually considered. In the latter case, prior to Pd-layer deposition, the support needs to be pre-treated either by Chemical Vapour Deposition (CVD) activation [5], or an Al_2_O_3_ suspension [23] or a sol-gel coating technique [21,24].

Numerous methods for depositing a thin Pd-layer onto porous supports have been investigated [25,26,27,28,29,30,31] and the technique most widely used is the electroless plating (ELP) owing to its various benefits [13,30,31]. Indeed, the hardness of the deposited layer and its strong adhesion to the support, the deposit uniformity on the complex geometry as well as the low preparation cost and the simplicity of the equipment and procedure can be considered as some of these benefits in the use of this technique [24,32].

Currently, the issue requiring to be overcome is related to the long-term stability of Pd-composite membranes under different operating conditions. To the best of our knowledge, there are few scientific papers dealing with the long-term test of Pd-based composite membranes [24,33,34,35,36]. For instance, Nair *et al*. [33] studied the permeating characteristics of Pd supported on alumina membrane, characterized by a 13 µm Pd-layer and reported a decrease of H_2_/N_2_ ideal selectivity after 300 h. Peters *et al*. [24] tested Pd–Ag supported on PSS in 50% H_2_/N_2_ at 19.6 bar for 2400 h at 350–450 °C and observed a gradual H_2_/N_2_ selectivity decrease from 3000 to 500. They attributed the leak growth to pinhole formation. Also Kulprathipanja *et al*. [35] observed a significant decrease in the H_2_/N_2_ ideal selectivity, testing 4 µm Pd–Cu supported onto a porous alumina membrane after 340 h. On the contrary, Yun *et al*. [36] reported a stable H_2_/N_2_ ideal selectivity of 9200 for 150 h at 460 °C by investigating an ultra-thin Pd composite membrane.

Consequently, the aim of this work is to evaluate the long-term stability, as well as estimating the life-time, of two different composite membranes, characterized by a thin Pd-layer supported onto alumina and PSS supports, under different operating conditions. Furthermore, comparisons with experimental data from the open literature are also made and further discussed.

## 2. Experimental

### 2.1. Membrane Preparation and Membrane Module Setup

Several methods have been developed for depositing a palladium layer onto porous substrates such as magnetron sputtering [25], spray pyrolysis [26], CVD [27,28], physical vapour deposition [29] and ELP [13,30,31]. In this work, ELP technique has been chosen for preparing both composite membranes owing to its important benefits such as the uniformity of the thin palladium layer, simplicity of the required equipment and low cost.

The first membrane consists of ~10 μm of Pd-layer deposited onto a PSS tubular support, with a mean pore size of about 2 µm and a porosity around 20%. The support was supplied by Mott Metallurgical Corporation (Farmington, MI, USA) having a 1.0 cm O.D. AISI 316L porous tube with a total length equal to 7.7 cm. The porous support was welded to two non porous AISI 316 L tubes (Industrial Welding Corporation, Manila, Philippines), one of which was closed.

This membrane was manufactured at RSE Spa (IT) laboratories and its preparation was carried out by using a similar procedure to Ma *et al*. [18]. This procedure consists of machine polishing of the support with a diamond paste to reduce surface roughness, support cleaning in an ultrasonic bath, support oxidation in an oven with air at 500 °C, support activation and successive deposition of the Pd-layer by ELP [37,38]. After deposition, the Pd/PSS membrane presents an active area equal to 24.2 cm^2^.

**Figure 1:**
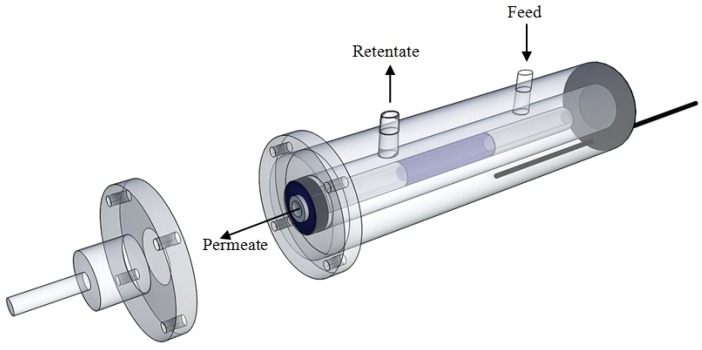
Scheme of membrane module housing the Pd/porous stainless steel (PSS) membrane.

The membrane module, used for housing the aforementioned membrane, is shown in Figure 1 and consists of a tubular stainless steel module (length 280 mm, internal dimater (i.d.) 20 mm), in which a graphite o-ring avoids the mixing of permeate and retentate streams.

**Figure 2:**
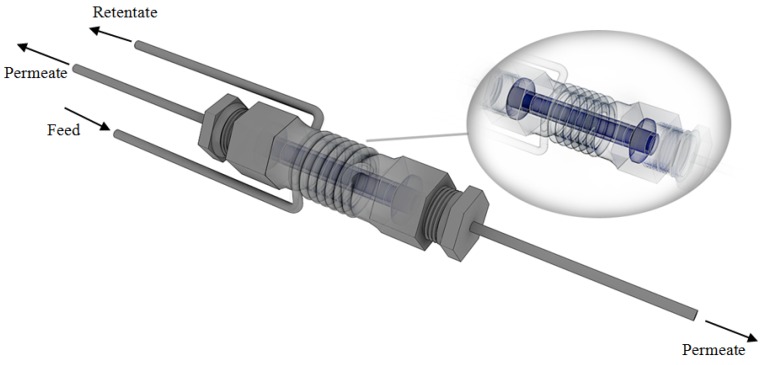
Scheme of membrane module housing the Pd/Al_2_O_3_ membrane.

The second membrane was manufactured at Nanjing University of Technology (Nanjing, China) by following the preparation procedure of Collins and Way [39]. It consists of ~7 μm Pd-layer deposited onto an Al_2_O_3_ support, supplied by Gao Q Funct. Mat. Company (Nanjing, China). The support shows a total length equal to 7.5 cm with an outer and inner diameter equal to 1.3 cm and 0.9 cm, respectively, and it is open at both ends. The active area of the Pd/Al_2_O_3_ membrane is about 17.2 cm^2^. In this case, two graphite o-rings are used to ensure that the permeate and retentate streams are not mixed with each other. In Figure 2, the scheme of the Pd/Al_2_O_3_ membrane module is shown.

### 2.2. Experimental Details

The experimental plant used for carrying out the long-term tests consists of: Brooks instruments 5850S mass-flow controllers, which regulate the gas feed flow rates, a membrane module and a bubble flow meter for measuring the gas permeating flux through the membrane. Both membranes were heated in a helium atmosphere. The investigated temperatures were in the range 350–450 °C for Pd/Al_2_O_3_ and 380–420 °C for Pd/PSS. The restricted processing temperature range of Pd/PSS is due to its temperature limits. In particular, the lower and upper temperature limits are 360 °C and 430 °C, respectively. The pressure in the retentate side was varied from 2.0 to 8.0 bar by means of a back pressure placed at the outlet side, while the pressure in the permeate side is kept constant at 1.0 bar without using any sweep gas.

The measurements are carried out for a period of around 2000 h.

Concerning the performance description of both Pd-based composite membranes, some equations have been defined as reported below:

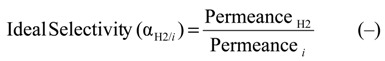
(1)

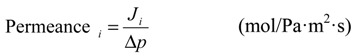
(2)
where *i* can be He, N_2_, H_2,_ CH_4_, CO_2_; *J_i_* the permeating flux of *i*-gas; and ∆*p* the trans-membrane pressure.

## 3. Result and Discussion

### 3.1. Permeation Measurements

**Figure 3:**
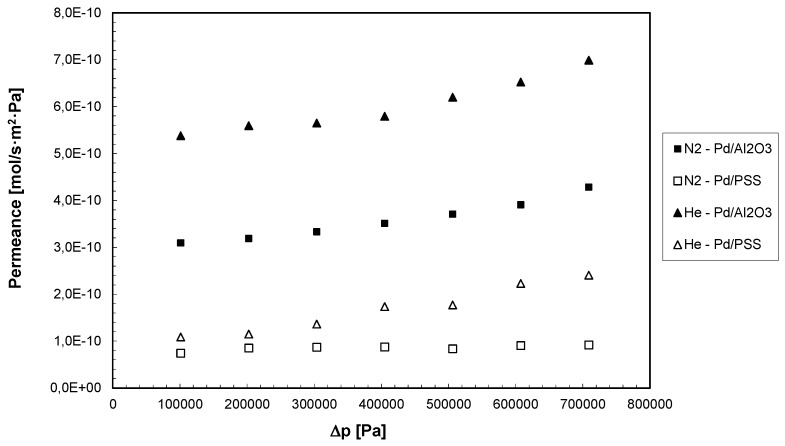
N_2_ and He permeances as a function of trans-membrane pressure for both Pd-based membranes at *T* = 400 °C.

Permeation tests with pure gases such as H_2_, N_2_, He, CH_4_, CO_2_ have been performed in the range of 200–800 kPa, by using both Pd-based composite membranes. Firstly, the permeation tests were performed with He and N_2_ for checking the presence of any defect in the palladium layer. In Figure 3, at 400 °C the permeance of N_2_ and He for both membranes as a function of the trans-membrane pressure is reported.

It appears evident that the permeance of each gas is increased by increasing the trans-membrane pressure, showing that both membranes are affected by pinholes or defects. These imperfections can be caused, among others, by the decomposition of some impurities present in the ELP solution and co-deposited onto porous supports with palladium particles [12]. As a comparison at each trans-membrane pressure investigated, the Pd/PSS membrane showed lower He and N_2_ permeance values than the Pd/Al_2_O_3_ membrane, illustrating that the first membrane is characterized by a lower presence of defects in the Pd-layer. The same behavior was found by carrying out the permeation tests with CH_4_ and CO_2_. Moreover as shown in Table 1, CO_2_ and CH_4_ permeances for both membranes are increased by increasing the trans-membrane pressure.

**Table 1:** CH_4_ and CO_2_ permeances as a function of the trans-membrane pressure difference for both Pd-based composite membranes at *T* = 400 °C.

∆ *p* (Pa)	Pd/Al_2_O_3_		Pd/PSS	
	CH_4_ Permeance	CO_2_ Permeance	CH_4_ Permeance	CO_2_ Permeance
	(mol/s·m^2^·Pa)	(mol/s·m^2^·Pa)	(mol/s·m^2^·Pa)	(mol/s·m^2^·Pa)
1.01 × 10^5^	6.27 × 10^−10^	7.28 × 10^−10^	2.73 × 10^−10^	3.52 × 10^−10^
2.02 × 10^5^	8.11 × 10^−10^	1.18 × 10^−9^	3.60 × 10^−10^	5.11 × 10^−10^
3.03 × 10^5^	9.91 × 10^−10^	1.36 × 10^−9^	4.01 × 10^−10^	5.75 × 10^−10^
4.04 × 10^5^	1.05 × 10^−9^	1.61 × 10^−9^	4.27 × 10^−10^	6.68 × 10^−10^
5.05 × 10^5^	1.09 × 10^−9^	1.68 × 10^−9^	4.46 × 10^−10^	7.08 × 10^−10^
6.06 × 10^5^	1.13 × 10^−9^	1.75 × 10^−9^	4.70 × 10^−10^	7.34 × 10^−10^
7.07 × 10^5^	1.17 × 10^−9^	1.86 × 10^−9^	4.89 × 10^−10^	7.41 × 10^−10^

As a further investigation, the influence of temperature was also evaluated for both membranes at ∆*p* = 1.0 bar. In particular, for Pd/PSS and Pd/Al_2_O_3_ membranes the temperature was varied between 380–420 °C and 350–450 °C, respectively. As a result, N_2_, He, CO_2_ and CH_4_ permeances are decreased by increasing temperature for both membranes. Therefore, as a global consideration, it was observed that, for each gas among He, N_2_, CO_2_ and CH_4_, the permeance was increased almost linearly by increasing the trans-membrane pressure (Figure 3 and Table 1) and by decreasing the temperature (not shown), indicating that the pure gas permeation through the membrane takes place by Knudsen diffusion and viscous flow through the defects [40].

### 3.2. Hydrogen Permeation Test

Recently, hydrogen transport through dense Pd-layer membranes has been extensively studied and it has been considered as a complex process that, generally, takes place by a solution-diffusion mechanism, which involves several steps, such as mass transfer, surface-reactions, bulk diffusion, *etc*.

Generally, the hydrogen permeating flux through the membrane can be expressed by the following equation:

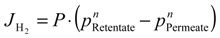
(3)
where *J*_H2_ is the hydrogen permeating flux through the membrane, *P* the hydrogen permeance, *p*_H2-retentate_ and *p*_H2-permeate_ are the hydrogen partial pressure in the retentate and permeate sides, respectively, and n the dependence factor of the hydrogen flux on the hydrogen partial pressure, generally in the range of 0.5–1. The n value is often used as an indicator for the rate-controlling step of the hydrogen permeation through the palladium (composite or not) membrane. Values equal to 0.5 (Sieverts-Fick’s law) indicate that the diffusion of atomic hydrogen through the dense metal layer is the rate-limiting step.

Deviations from this value can be attributed to a several factors such as defects in the Pd-layer, palladium surface contamination or transport resistance of the substrate material. However, for a thin membrane (<1 µm) the surface-reaction is the controlling step and *n* is equal to 1.

The hydrogen permeance is influenced by temperature by the Arrhenius law:

*P* = *P*° · exp (− *Ea*/*RT* ) (4)

where *P*°, *Ea*, *R* and *T* are the pre-exponential factor, apparent energy activation, universal gas constant and absolute temperature, respectively.

Generally, in order to establish the membrane permeation characteristics, *P*°, *Ea* and *n* need to be estimated. Therefore, in this work hydrogen permeation measurements at different temperature and trans-membrane pressure are required. 

**Figure 4:**
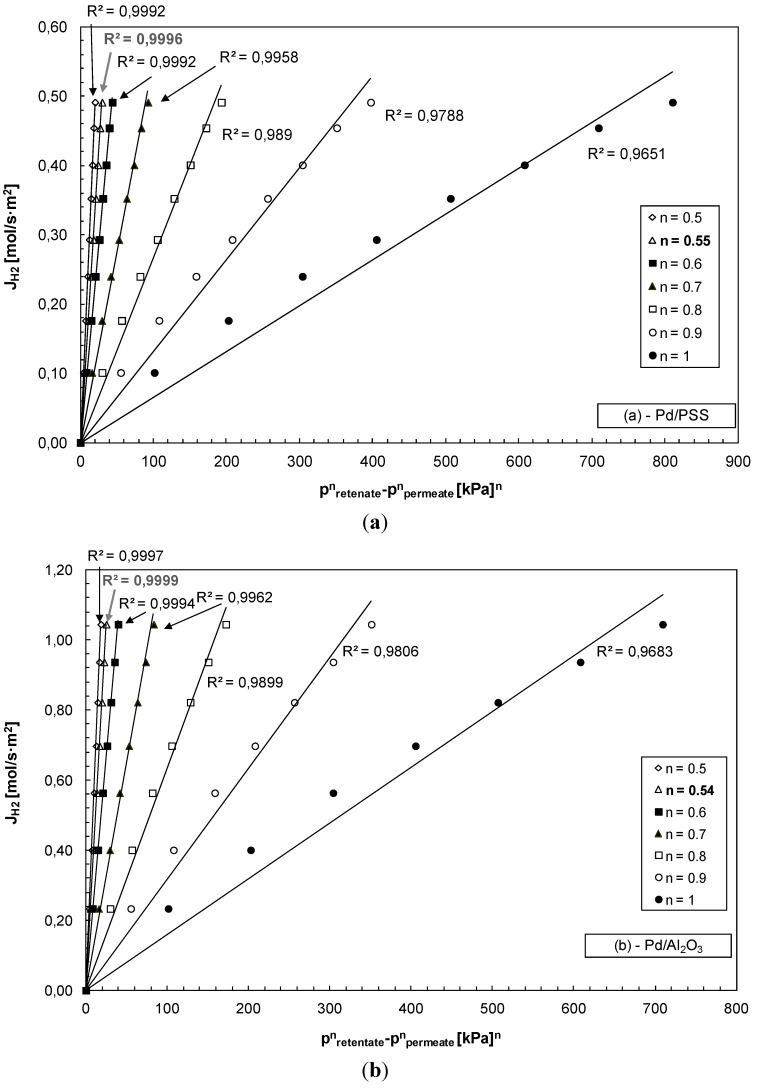
H_2_ permeating flux as a function of the trans-membrane pressure at different “*n*” values; *T* = 400 °C, (**a**) Pd/PSS and (**b**) Pd/Al_2_O_3_ membrane.

At 400 °C and by varying the trans-membrane pressure, hydrogen permeating flux through the Pd/PSS is shown in Figure 4a and through the Pd/Al_2_O_3_ membrane in Figure 4b by considering various “*n*” values. Depending on each “*n*” considered, the highest linear regression factors (*R*^2^) were found to be *n =* 0.55 for Pd/PSS and *n =* 0.54 for Pd/Al_2_O_3_. Therefore, the transport of hydrogen through both composite membranes is mainly limited by diffusion through the palladium bulk. Nevertheless, the deviation from the 0.5 value suggests that hydrogen flux does not depend only on atomic diffusion through palladium, but is influenced by other factors such as the presence of impurities on the membrane surface which could hinder hydrogen dissociation [41].

**Figure 5:**
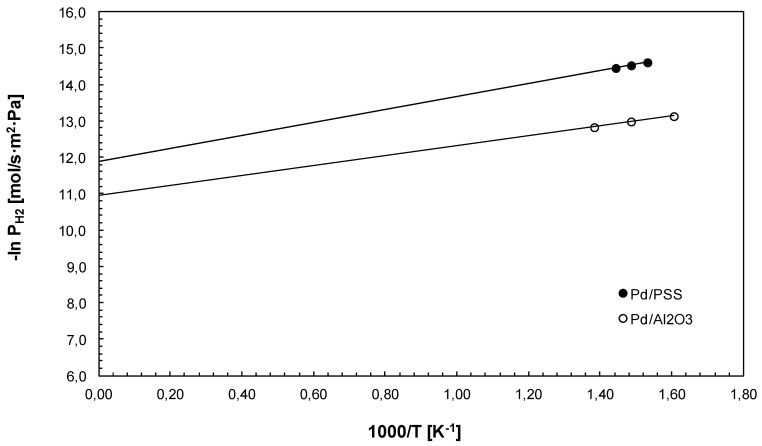
Arrhenius plot for pure hydrogen permeation tests using Pd/PSS and Pd/Al_2_O_3_ membranes at ∆*p* = 1 bar.

Furthermore, as shown in Figure 5 the temperature was varied for estimating *Ea* and *P*° for both membranes by using an Arrhenius plot of the hydrogen permeance against the reciprocal temperature. As a result, *Ea* equal to 14.7 kJ/mol and 11.4 kJ/mol and *P*° equal to 6.85 × 10^−6^ mol/s·m^2^·Pa and 1.77 × 10^−5^ mol/s·m^2^·Pa were evaluated for Pd/PSS and Pd/Al_2_O_3_ membranes, respectively.

Table 2 summarizes a comparison between the *Ea* and *n* values determined in this work with respect to other similar data found in the specialized literature.

As shown, the values of *Ea* are in good agreement with those reported by other researchers. For instance, Wang *et al*. [42] found 7.1 kJ/mol, Tong *et al*. [43] detected 16.2 kJ/mol and Zahedi *et al*. [44] reported a similar value to the one obtained with the Pd/PSS membrane of this work by using a Pd composite supported onto PSS modified with WO_3_. Regarding the n value, deviation from 0.5 was also found by other authors [31,39,43,45,46,47,48].

**Table 2:** Comparison of *Ea* and *n* values determined for both Pd/PSS and Pd/Al_2_O_3_ membranes with respect to similar data on Pd-based membranes reported in the literature.

Membrane	Layer of Pd (μm)	*Ea* (kJ/mol)	*n*	References
Pd/WO_3_/PSS	12	14.7	0.5	[44]
Pd/Al_2_O_3_	8.5	5.7	0.778	[45]
Pd/ZrO_2_/PSS	10	7.1	0.5	[42]
Pd/CeO_2_/PSS	6	17.3	0.85	[43]
Pd/Al_2_O_3_	11.4	8.9	0.58	[39]
Pd/SiO_2_/PSS	6	24 ^a^	1	[46]
Pd/FeCr/PSS	11	15.03	0.5	[49]
Pd/Al_2_O_3_	4.5	18.3	1	[47]
Pd/Al_2_O_3_	5	7.0	1	[31]
Pd/MPSS	20	16.4	0.60	[48]
Pd/Al_2_O_3_/PSS	15	20.6	0.5	[7]
Pd/PSS	10	14.7	0.55	This work
Pd/Al_2_O_3_	7	11.4	0.54	This work

^a^ Calculations based on permeation data in the literature.

### 3.3. Membrane Performance

**Figure 6:**
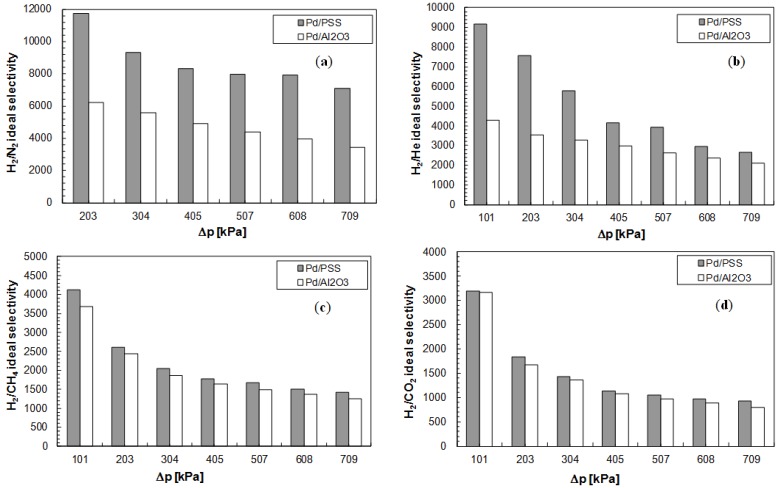
Ideal selectivities as a function of the trans-membrane pressure at *T* = 400 °C for Pd/PSS and Pd/Al_2_O_3_ membranes. (**a**) α_H2/N2_; (**b**) α_H2/He_; (**c**) α_H2/CH4_; (**d**) α_H2/CO2_.

By considering the permeance values at different trans-membrane pressures of each aforementioned gas, it is possible to evaluate the performance of both Pd-based membranes in terms of hydrogen over the other gases ideal selectivities. Figure 6a–d reports the comparison of the ideal selectivities as a function of trans-membrane pressure for both Pd/PSS and Pd/Al_2_O_3_ membranes at *T* = 400 °C.

It appears clear that, in all analyzed cases, the ideal selectivity of hydrogen with respect to other gases is decreased by increasing the trans-membrane pressure. This behavior can be explained by taking into account the possible permeation mechanisms of each gas through the membrane. In particular, for He, N_2_, CO_2_ and CH_4_, it has been observed that the permeating flux of each gas, taking place by Knudsen diffusion and viscous flow through the defects [40], is increased almost linearly by increasing the trans-membrane pressure. On the contrary, hydrogen permeates mainly by a solution-diffusion mechanism and its flux depends on pressure with a factor equal to 0.55 for the Pd/PSS and 0.54 for the Pd/Al_2_O_3_ membrane. Consequently, with rising pressure the increment of the permeating flux of each gas (He, N_2_, CH_4_ and CO_2_) is higher than that of hydrogen. As a result, the ideal selectivity of hydrogen decreases with increasing trans-membrane pressure.

The H_2_/N_2_ and H_2_/He ideal selectivities illustrated in Figure 6a,b were evaluated at the beginning of the experimental campaign, showing that the Pd/PSS membrane is characterized by higher ideal selectivities than Pd/Al_2_O_3_ membrane. On the contrary, the H_2_/CH_4_ and H_2_/CO_2_ ideal selectivities have been calculated after temperature variations, from 380 to 420 °C for Pd/PSS membrane and from 350 °C to 450 °C for Pd/Al_2_O_3_ one. Therefore, from Figure 6c,d, it is evident that there has been a deterioration of both membranes with respect to what reported in Figure 6a,b. In particular, the loss of performance in terms of ideal selectivity for the Pd/PSS membrane is shown to be larger than for the Pd/Al_2_O_3_ membrane.

In Table 3, the Pd/PSS and Pd/Al_2_O_3_ performance in terms of hydrogen flux, permeance and H_2_/N_2_ ideal selectivity are compared with similar data taken from the open literature.

**Table 3:** Comparison with scientific literature data.

Membrane type	Preparation method	Pd layer (μm)	*T* (°C)	∆ *p* (Pa)	H_2_ flux (mol/s·m^2^)	H_2_ permeance (mol/s·m^2^·Pa)	α(H_2_/N_2_) (–)	References
Pd/CeO_2_/PSS	ELP-CVD	6	500	100,000	0.235	–	14 ^b^	[50]
Pd/γ-Al_2_O_3_/Al_2_O_3_	ELP	5	300	400,000	–	1.4 × 10^−6^	1000	[51]
Pd/ZrO_2_/PSS	ELP	23	400	100,000	0.0734	5.2 × 10^−4 a^	320	[5]
Pd/γ-Al_2_O_3_/Al_2_O_3_	ELP	2.4	500	100,000	–	3.9 × 10^−6^	32,500	[52]
Pd/Ni-SiO_2_/PSS	CVD	–	450	42,000	–	6.4 × 10^−6^	6100	[53]
Pd/YSZ/PSS	ELP	28	450	30,000–40,000	0.01–0.06	4.5 × 10^−4 a^	∞	[54]
Pd/γ-Al_2_O_3_/Al_2_O_3_	ELP	2.6	370	400,000	–	4.8 × 10^−7^	3000	[33]
Pd/γ-Al_2_O_3_/Al_2_O_3_	ELP	1	400	75,000	–	6.7 × 10^−6^	23	[55]
Pd/SiO_2_/PSS	CVD	6	500	50,000	0.133	2.7 × 10^−6^	450	[46]
Pd/Al_2_O_3_	CVD	2	300	30,000	–	3.3 × 10^−6^	5000	[56]
Pd/Fe_2_O_3_/PSS	ELP	22	450	100,000	0.0853	2.7 × 10^−4 a^	∞	[13]
Pd/γ-Al_2_O_3_/Al_2_O_3_	ELP	6	480	100,000	–	2.6 × 10^−6^	2100^ b^	[31]
Pd/Al_2_O_3_	CVD	1	450	68,000	–	2.1 × 10^−6^	780	[28]
Pd/Al_2_O_3_	ELP	0.9	460	199,000	–	3.1 × 10^−6^	1200	[36]
Pd/PSS	ELP	20	350	100,000	–	5 × 10^−7^	5000	[40]
Pd/NaAZ/PSS	ELP	19	450	50,000	0.0790	1.1 × 10^−3 a^	608	[57]
Pd/PSS	ELP	10	400	200,000	0.176	8.7 × 10^−7^	11,800	This work
Pd/Al_2_O_3_	ELP	7	400	100,000	0.233	2.3 × 10^−6^	7500	This work

^a^ this value is given in mol/s·m^2^·Pa^0.5^; ^b^ H_2_/Ar Ideal Selectivity.

A rigorous quantitative comparison is quite difficult to carry out owing to the many different parameters involved such as the preparation method, Pd-layer, membrane type as well as the operating conditions (temperature and pressure range). From a qualitative point of view, it is evident that membranes, characterized by a thinner Pd-layer, show higher hydrogen permeating fluxes; nevertheless, their H_2_/N_2_ ideal selectivity is far from the ideal value of infinite, probably because of the presence of pinholes or defects. On the contrary, membranes having a thicker Pd-layer present lower hydrogen permeating flux but higher ideal selectivity. In particular, the membranes produced by Rothenberger *et al*. [13] exhibited an infinite ideal selectivity, working at ~0.3 bar as trans-membrane pressure. Nevertheless, these authors stated that this ideal-selectivity is not ensured at higher pressure. Indeed, when they increased the pressure from 5.0 to 28.0 bar, the ideal selectivity dropped from a value of 135 to 12.

However, as shown in Table 3 the performance of both Pd/PSS and Pd/Al_2_O_3_ membranes from this work are coherent with data from the open literature. In particular, the results indicate that both membranes present high hydrogen permeating fluxes and ideal selectivities.

**Figure 7:**
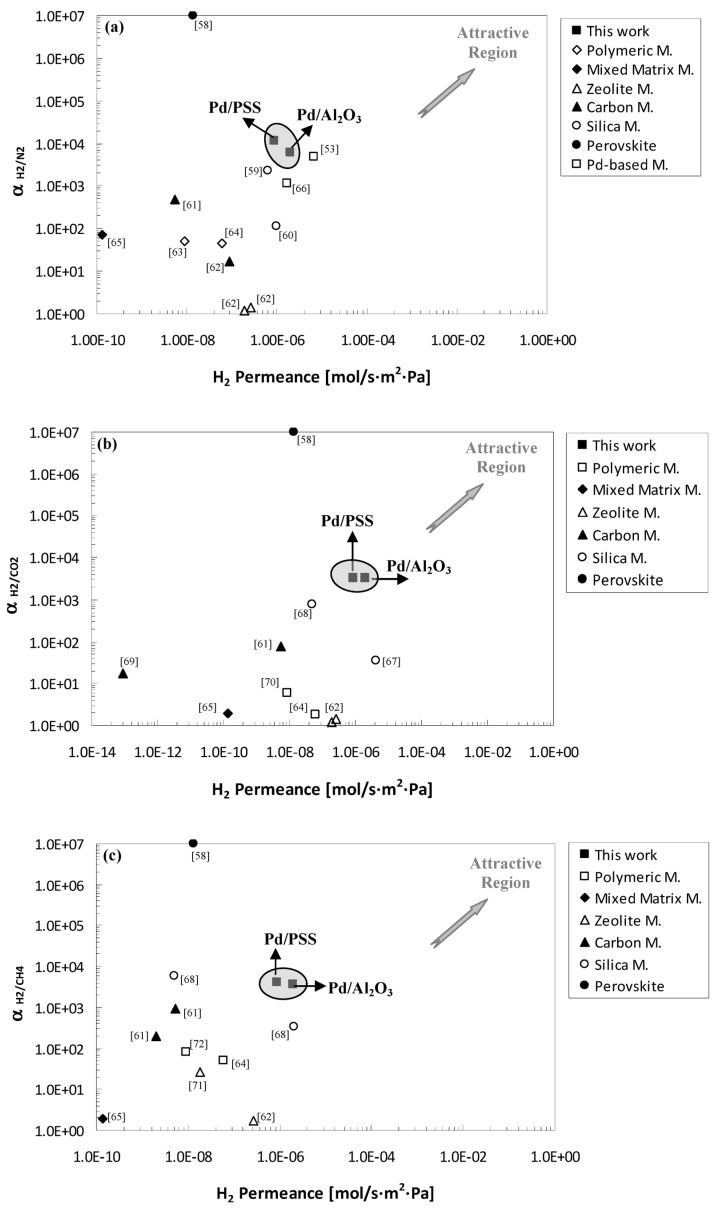
Comparison with scientific data. (**a**) α_H2/N2_; (**b**) α_H2/CO2_; and (**c**) α_H2/CH4_ *vs.* H_2_ Permeance [53,58,59,60,61,62,63,64,65,66,67,68,69,70,71,72].

As a further comparison, Figure 7a–c shows the ideal selectivity of H_2_/N_2_, H_2_/CO_2_ and H_2_/CH_4_ against hydrogen permeance. In particular, the performance of the membranes of this work were compared to literature data obtained by using other kind of membranes such as polymeric, silica, carbon, mixed matrix and zeolite.

As well known, a trade-off exists between permeance and selectivity. Indeed, more permeable membranes are generally less selective and *vice versa*. In this contest, the efforts of research are devoted to produce membranes highly permeable and selective towards H_2_.

By referring to Figure 7a–c, the best performance are expected in the upper right hand corner. It is evident that both the Pd/PSS and Pd/Al_2_O_3_ membranes studied in this work showed a good compromise between ideal selectivity and H_2_ permeance with respect to other referenced membranes. For instance, perovskite membranes [58] present very high ideal selectivity but low H_2_ permeance working at high temperature. An opposite trend is shown by zeolite membranes, while comparable results to Pd-based membranes of this work were realized by using silica membranes [59,68]. Nevertheless, the CVD technique used for manufacturing these membranes is quite expensive.

### 3.4. Membrane Long-Term Stability and Life Time Estimation

**Figure 8:**
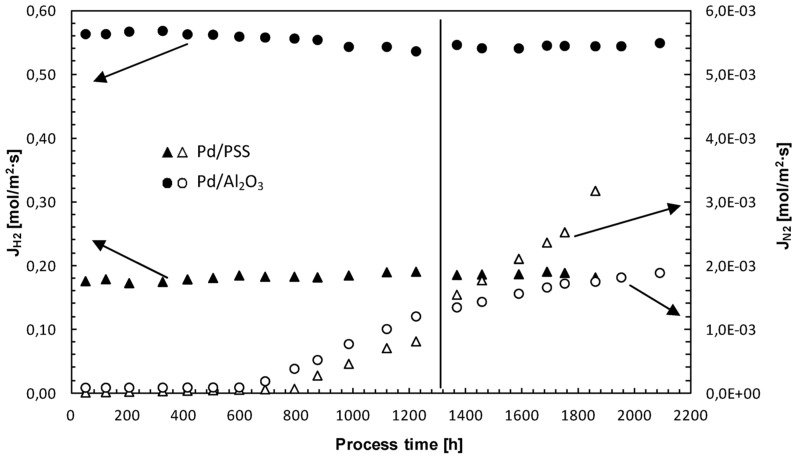
H_2_ and N_2_ permeating flux through both Pd/PSS and Pd/Al_2_O_3_ membranes as a function of process time.

The long-term H_2_ permeance and H_2_/N_2_ ideal selectivity stability of Pd-based composite membranes represent an important key point for their real commercial application. Therefore, long-term stability tests were carried out by monitoring both composite membranes over a period of around 2000 h through permeation measurements with H_2_ and N_2_. At 400 °C and ∆*p* = 200 kPa, H_2_ and N_2_ permeating fluxes through both Pd/PSS and Pd/Al_2_O_3_ membranes are given as a function of process time, Figure 8.

It is evident that hydrogen permeating flux through both membranes can be considered relatively stable for long-time operation under different operating conditions.

Concerning the N_2_ flux, it can be considered constant up to 600 h and, then, increases progressively as a function of time. Up to 600 h, the operating temperature was kept constant at 400 °C. Successively, for performing permeation tests, it was varied from 380 to 420 °C for the Pd/PSS membrane and from 350 to 450 °C for the Pd/Al_2_O_3_ membrane. This temperature variation probably caused some changes in both Pd-based membrane structures [13,73]. In particular, as reported by Guazzone and Ma [73], it is possible that, at 400–450 °C, new pinholes could be formed owing to the sintering of small Pd clusters. More recently, Augustine *et al*. [74] evaluated the long-term stability of various Pd/PSS membranes, also in the presence of steam and synthetic WGS mixture at 400–450 °C. These authors noticed that, although a decrease of selectivity was evidenced during the time, final values remained rather high (above 1000). In this work, however, the H_2_/N_2_ ideal selectivity of the Pd/PSS membrane dropped from 11700 to 55 after 1800 h of operating time, while it decreased from 6200 to 310 after 2100 h for Pd/Al_2_O_3_. The dramatic decrease of H_2_/N_2_ ideal selectivity for the Pd/PSS membrane could be attributed to the presence of some contaminants, rather than to the growth of pre-existing pinholes.

**Figure 9:**
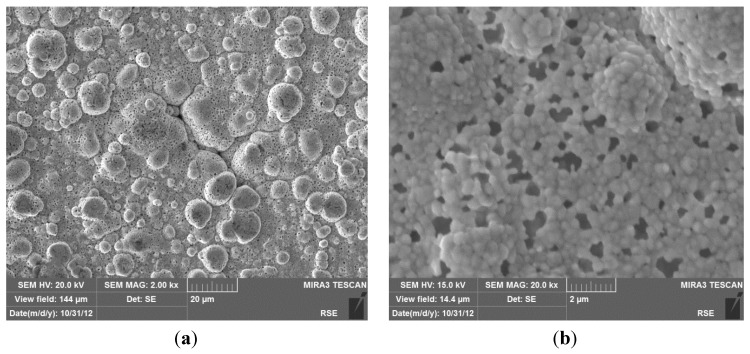
SEM images with secondary electrons of the Pd/PSS membrane surface at the end of the tests. (**a**) Typical morphology of Pd-clusters; (**b**) Presence of pinholes on the Pd-surface.

To investigate the causes of membrane degradation, the Pd/PSS membrane was analyzed by Scanning Electron Microscope (SEM). SEM images of the membrane surface are shown in Figure 9, which highlight the characteristic cauliflower morphology of Pd-clusters and the presence of pinholes on the whole surface, up to micron size. The formation of new pinholes in the palladium layer was probably determined by sulphur release from graphite seals, as previously investigated by the same authors utilizing a twin Pd/PSS membrane used in this work [75].

## 4. Conclusions

Two composite membranes, Pd/PSS and Pd/Al_2_O_3_, constituted by thin palladium layers deposited onto porous supports via ELP have been studied in the temperature range of 350–450 °C and from 100 to 800 kPa as retentate pressure.

The experimental measurements illustrated that, at 400 °C, both types of membranes showed interesting H_2_/other gases ideal selectivities and hydrogen flux combined to long-term stability up to 600 h. Nevertheless, when the temperature was varied H_2_/N_2_ ideal selectivity decreased as a function of process time. In particular, H_2_/N_2_ ideal selectivity of the Pd/PSS membrane dropped from 11700 to 55 after 1800 h, whereas it decreased from 6200 to 310 after 2100 h for the Pd/Al_2_O_3_ membrane. This decrease was attributed for the Pd/PSS membrane to the formation of new pinholes in the palladium layer determined by sulphur release from graphite seals that, most likely, contributed to the degradation of both membranes.
